# Medical News

**Published:** 1853-06

**Authors:** 


### MEDICAL NEWS.

UNIVERSITY OF PENNSYLVANIA.—Dr. JOSEPH LEIDY, of Philadelphia, has been appointed Professor of Anatomy in this Institution, in place of Dr. Horner, deceased.

We congratulate the friends of this venerable school in the selection of one so eminently qualified to occupy the post of the lamented Horner. His talents and industry cannot fail to win and secure the admiration of those who may attend upon his teachings.

ST. JOSEPH'S HOSPITAL.—Dr. WM. B. PAGE has been appointed one of the Surgeon's in place of Dr. Horner, deceased.

By a late paper, we learn the arrival of Drs. WOOD and BACHE in Paris.

Drs. MOTT of New York, and WARREN of Boston, have been elected members of the *Academie de Medicine* of Paris.

The seeds of Parsley and Celery have been shown to have a decided influence over malarious fevers, although their operation is not equal to Quinine.

It is announced that the second volume of Pereira's Materia Medica will be arranged for publication in England this summer.

Dr. WILLIS G. EDWARDS, Professor of Chemical Medicine and Pathological Anatomy in the Medical Department of the St. Louis University, resigned his chair on the 14th of March, on account of his continued ill health and his consequent anticipated removal from the city.

